# Influence of Temporal and Spatial Fluctuations of the Shallow Sea Acoustic Field on Underwater Acoustic Communication

**DOI:** 10.3390/s22155795

**Published:** 2022-08-03

**Authors:** Zhichao Lv, Libin Du, Huming Li, Lei Wang, Jixing Qin, Min Yang, Chao Ren

**Affiliations:** 1 College of Ocean Science and Engineering, Shandong University of Science and Technology, Qingdao 266590, China; dulibin@sdust.edu.cn (L.D.); lihuming78@163.com (H.L.); wangleiimg@foxmail.com (L.W.); 2 State Key Laboratory of Acoustics, Institute of Acoustics, Chinese Academy of Sciences, Beijing 100190, China; qjx@mail.ioa.ac.cn; 3 North China Sea Marine Technical Support Center, State Oceanic Administration, Qingdao 266061, China; 4 Acoustic Science and Technology Laboratory, Harbin Engineering University, Harbin 150001, China; qingtianxiayu@hrbeu.edu.cn

**Keywords:** underwater acoustic communication, channel characteristics, spatiotemporal fluctuation

## Abstract

In underwater acoustic communication (UAC) systems, the channel characteristics are mainly affected by spatiotemporal changes, which are specifically manifested by two factors: the effects of refraction and scattering caused by seawater layered media on the sound field and the random fluctuations from the sea floor and surface. Due to the time-varying and space-varying characteristics of a channel, the communication signals have significant variations in time and space. Furthermore, the signal shows frequency-selective fading in the frequency domain and signal waveform distortion in the time domain, which seriously affect the performance of a UAC system. Techniques such as error correction coding or space diversity are usually adopted by UAC systems to neutralize or eliminate the effects of deep fading and signal distortion, which results in a significant waste of limited communication resources. From the perspective of the sound field, this study used experimental data to analyze the spatiotemporal fluctuation characteristics of the signal and noise fields and then summarized the temporal and spatial variation rules. The influence of the system then guided the parameter configuration and network protocol optimization of the underwater acoustic communication system by reasonably selecting the communication signal parameters, such as frequency, bandwidth, equipment deployment depth, and horizontal distance.

## 1. Introduction

At present, using sound waves is the only method for transmitting data over long distances in seawater. Underwater acoustic communication has become an indispensable part of data transmission technology for exploring, developing, and protecting the ocean. The complexity of UAC systems is mainly manifested in the time-varying and space-varying channels. The signal-to-noise ratio (SNR) as a measurement parameter of system performance design is also an important indicator for evaluating the quality of underwater acoustic communication. The spatiotemporal variation range of the SNR can be used to describe the spatiotemporal fluctuation characteristics of underwater acoustic communication signals.

Due to the complexity and time variation of the marine environment, the SNR of communication signals varies widely in time and space. Therefore, the degeneration in the UAC system performance would be caused by two reasons: the difficulties in optimizing the synchronization signal detection threshold and determining the location of the UAC system equipment. However, from the perspective of the sound field, it is possible to describe the spatiotemporal variation from the signal field and noise field, and then analyze the fluctuation characteristics of the underwater acoustic communication channel. Environmental factors, such as ocean currents, tides, and internal waves, with large spatial-temporal fluctuations have not been considered.

Since the 1950s, researchers have gradually paid attention to signal interference in shallow-water sound fields, and they mainly analyzed the spatial-temporal characteristics of sound fields based on ray acoustic theory and normal wave theory [1,2,3]. Ray acoustics researchers focused on the Loe mirror effect in optics, which assumes that the interfering sound rays are approximately parallel, and they derived a partially analytical solution and discovered vertical distribution characteristics of the sound field [4]. In the 1980s, in view of the normal wave theory and far-field assumption, scholars analyzed the interference sound field and put forward the conception of waveguide invariants. However, previous theoretical analyses of the interference phenomenon, which are based on restricted scenarios and simplified assumptions, were detrimental to the establishment of a universal analysis model. This study mainly focused on the influence of short-range shallow sea interference on signal fluctuations. Compared with the normal wave method, the ray acoustic method is superior given its clear concept and simple calculation; therefore, the ray acoustic method was adopted to analyze the spatial distribution of the sound field.

From the perspective of the sound field, the influencing factors of the spatial variation range of the SNR are not only the spatial-temporal distribution characteristics of the signal field caused by the signal interference and interface fluctuations but also the spatial-temporal distribution characteristics of the system noise field. The operating performance of a UAC system is significantly affected by the noise of the system and the marine environment. The marine environmental noise was first measured in two studies [5,6]. After analyzing a large amount of measured data, it was found that marine environmental noise is mainly composed of wind-induced noise, ship noise, and biological noise; reference [7] gives a marine environmental noise spectrum and notes that the low-frequency noise components mainly come from the machinery of ships, while high-frequency noise is mainly wind-induced wave noise. Due to the continuous development of signal acquisition technology and the increase in marine research investment, research on marine environmental noise has become a popular topic of discussion [8,9,10,11,12,13,14,15]. On the basis of different sound field propagation theories, researchers proposed various marine environmental noise models. The classic models include the C/S model, K/I fast-field model, and P/K model [16,17,18]. Using these models, scholars have conducted in-depth studies on calculation accuracy, calculation speed, orientation, and boundary conditions, and concluded a series of results, which promoted the development and application of exploration of the noise field [19,20,21,22]. Zhou Jianbo et al. considered wind-induced waves as the noise source and used the transmission theory method instead of the traditional Monte Carlo method to construct a noise field model. They analyzed the spatial noise distribution and concluded that high-frequency noise had fluctuations in intensity at the offshore surface [23]. Avrashi, G. et al. considered the problem of carrier frequency offset estimation in OFDM underwater acoustic communication and analyzed the causes of changing environmental impacts [24]. Z.L. et al. analyzed wave fluctuation on underwater acoustic communication using measured data collected with USV [25]. X.Z. et al. used quantile–quantile (Q-Q) plots to analyze real marine environmental data, interpreting the impulsive property of ocean ambient noise in shallow waters [26]. X.Z. et al. applied Loffeld’s bistatic formula to SAS image processing, which provided a more accurate approximation of the spectrum compared to that based on phase center approximation [27]. An, J. et al. propose underwater acoustic (UWA) communications using a generalized sinusoidal frequency modulation (GSFM) waveform, which makes full use of the time and frequency variation laws of the marine environment in experimental data [28]. Zhang, Y. et al. proposed a deep-learning-based orthogonal frequency division multiplexing receiver for underwater acoustic communications to process marine environmental data through neural networks [29].

The stratum structure of the ocean space determines the multi-channel coherent structure characteristics of the ocean sound field. The motion of the transmitter end, the interface, and the receiving sensor affect the spatiotemporal fluctuation characteristics of the sound field, which shows that the channel response function is time-varying and space-varying. The feature of time-varying and space-varying channels is the key point to manage to achieve effective and stable UAC systems. In this study, the signal field and noise field were evaluated by establishing a model and obtaining data through experiments, and the spatial-temporal distribution of signals was summarized to provide theoretical support for the design of UAC systems.

## 2. Spatial Distribution Characteristics of the Signal and Noise Fields

### 2.1. Investigation of the Spatial Distribution of the Signal Field

In a uniformly shallow sea, the characteristics of the medium do not change significantly with the depth; therefore, the sound field can be studied using ray theory. The accuracy of ray theory for calculating the sound field is correlated with the number of sound rays examined in the study. The larger the number of sound rays considered, the less the sound rays reflected from the bottom of the water contribute to the sound field. Thus, the direct sound and the first-order reflection rays of the sea surface are mainly considered. The sound pressure field is

(1) $$p\left(r,z,t\right)=\frac{1}{R_{00}}exp\left[i\left(kR_{00}-\omega t\right)\right]-G_{10}\frac{1}{R_{10}}exp\left[i\left(kR_{10}-\omega t\right)\right]$$

The direct sound path $R_{00}=\sqrt{r^{2}+{\left(z-z_{s}\right)}^{2}}$ and the first surface reflection sound path $R_{10}=\sqrt{r^{2}+{\left(z+z_{s}\right)}^{2}}$, where r is the horizontal distance between the sound source and the receiving hydrophone and $z_{s}$ is the depth of sound source placement; $G_{10}$ is the absolute value of the surface reflection coefficient. Separating out the time variable gives

(2) $$p\left(r,z\right)=\frac{1}{R_{00}R_{10}}exp\left(ikR\right)\left\{R_{10}exp\left[ik\left(R_{00}-R\right)\right]-G_{10}R_{00}exp\left[ik\left(R_{10}-R\right)\right]\right\}$$

where

(3) $$R_{00}-R=\sqrt{r^{2}+z^{2}}\left(\sqrt{1+\frac{z_{s}^{2}-2z_{s}z}{r^{2}+z^{2}}}-1\right)$$

When the value of r is greater than 3 times the depth of the sea, i.e., $\left(z_{s}^{2}-2z_{s}z\right)/\left(r^{2}+z^{2}\right)\ll 1$, Formula (3) can be approximated:

(4) $$R_{00}-R\approx \;\sqrt{r^{2}+z^{2}}\left(\sqrt{1+2\cdot \frac{z_{s}^{2}-2z_{s}z}{2\left(r^{2}+z^{2}\right)}+{\left[\frac{z_{s}^{2}-2z_{s}z}{2\left(r^{2}+z^{2}\right)}\right]}^{2}}-1\right)=\frac{z_{s}^{2}-2z_{s}z}{2\left(r^{2}+z^{2}\right)}$$

Similarly:

(5) $$R_{00}-R\approx \;\frac{z_{s}^{2}+2z_{s}z}{2\left(r^{2}+z^{2}\right)}$$

Substituting (4) and (5) into Equation (2), we obtain

(6) $$\begin{array}{ll} p=\frac{1}{R_{00}R_{10}}exp & \left[ik\left(R+\frac{z_{s}^{2}}{2\sqrt{r^{2}+z^{2}}}\right)\right]\\ & \times \{\left(R_{10}-G_{10}R_{00}\right)cos\left[k\left(\frac{z_{s}z}{\sqrt{r^{2}+z^{2}}}\right)\right]\\ & -i\left(R_{10}-G_{10}R_{00}\right)sin\left[k\left(\frac{z_{s}z}{\sqrt{r^{2}+z^{2}}}\right)\right]\} \end{array}$$

Then, the mean square sound pressure in the sound field is

(7) $$\begin{array}{ll} \bar{p^{2}} & =\frac{1}{2}\frac{1}{{\left(r^{2}+z^{2}+z_{s}^{2}\right)}^{2}-4z_{s}^{2}z^{2}}[\left(1+G_{10}^{2}\right)\left(r^{2}+z^{2}+z_{s}^{2}\right)+2\left(1-G_{10}^{2}\right)z_{s}z\\ & -2G_{10}\sqrt{{\left(r^{2}+z^{2}+z_{s}^{2}\right)}^{2}-4z_{s}^{2}z^{2}}cos\left(\frac{2kz_{s}z}{\sqrt{r^{2}+z^{2}}}\right)] \end{array}$$

When focusing on the sound field distribution in the central area of the water body, there is an approximate value of $z\approx z_{s}/2$; after substitution into Equation (7), this gives

(8) $$\begin{array}{ll} \bar{p^{2}} & =\frac{1}{2}\frac{1}{{\left(r^{2}+\frac{5}{4}z_{s}^{2}\right)}^{2}-z_{s}^{4}}[\left(1+G_{10}^{2}\right)\left(r^{2}+\frac{5}{4}z_{s}^{2}\right)+\left(1-G_{10}^{2}\right)z_{s}^{2}\\ & -2G_{10}\sqrt{{\left(r^{2}+\frac{5}{4}z_{s}^{2}\right)}^{2}-z_{s}^{4}}cos\left(\frac{2kz_{s}z}{\sqrt{r^{2}+z^{2}}}\right)] \end{array}$$

If the signal is a broadband signal, the following formula can be obtained:

(9) $$\bar{{\bar{p^{2}}}_{\Delta f}}=\frac{1}{\Delta f}\int_{f_{0}-0.5\Delta f}^{f_{0}+0.5\Delta f}\bar{p{\left(f\right)}^{2}}df$$

Further derivation can be written as follows:

(10) $$\begin{array}{ll} \bar{p^{2}} & =\frac{1}{2}\frac{1}{{\left(r^{2}+\frac{5}{4}z_{s}^{2}\right)}^{2}-z_{s}^{4}}[\left(1+G_{10}^{2}\right)\left(r^{2}+\frac{5}{4}z_{s}^{2}\right)+\left(1-G_{10}^{2}\right)z_{s}^{2}\\ & -2G_{10}\sqrt{{\left(r^{2}+\frac{5}{4}z_{s}^{2}\right)}^{2}-z_{s}^{4}}\frac{\sin\theta }{\theta }cos\left(\frac{2kz_{s}z}{\sqrt{r^{2}+z^{2}}}\right)] \end{array}$$

where $\theta =2\pi z_{s}z\cdot \Delta f/\left(c\sqrt{r^{2}+z^{2}}\right)$ and $k_{0}=2\pi f_{0}/c$. It is worth noticing that when the signal has a single frequency, i.e., Δf approximates 0, sinθ/θ approaches 1; furthermore, when the signal bandwidth increases, θ increases and sinθ/θ approaches 0. Given this, when the signal has sufficient bandwidth, the fluctuation of the sound field of the signal can be effectively smoothed. When $z\approx z_{s}/2$, the approximation can be given as

(11) $$\theta \approx \frac{\pi z_{s}^{2}\cdot \Delta f}{c\sqrt{r^{2}+z^{2}}}\approx \frac{\pi }{c}\frac{z_{s}^{2}}{r}\cdot \Delta f$$

According to Formula (11), it can be obtained that the fluctuation range of the signal is proportional to the distance r at the transmitting and receiving ends and inversely proportional to the center frequency f, the bandwidth Δf, and the modem depth $z_{s}$.

#### 2.1.1. Simulation Testing

In order to verify the theoretical distribution of the signal field, the signal sound intensity fluctuations of different center frequencies, bandwidths, and horizontal distances of the transceiver were simulated and examined.

1. Simulation of the signal interference at different center frequencies

Simulation parameters: the seabed was absolutely hard and flat; water depth: 100 m; sound source deployment depth: 95 m (near the seabed); horizontal distance of the transmitting and receiving end: 200 m; signal bandwidth: 10 Hz; frequencies were 500 Hz, 2 kHz, and 5 kHz.

**Figure 1:**
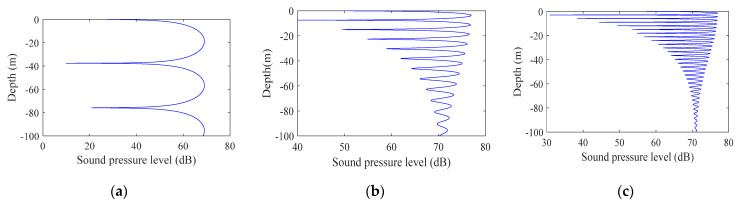
Signal interferograms at different frequencies: (**a**) 500 Hz, (**b**) 2000 Hz, and (**c**) 5000 Hz.

Figure 1 shows the signal interference diagrams at different frequencies. The frequencies from (a) to (c) were 500 Hz, 2 kHz, and 5 kHz. The abscissa is the signal sound pressure level and the ordinate is the depth. Comparing the figures, it can be seen that as the center frequency of the signal doubled, the vertical fluctuation range of the sound intensity decreased by a factor of half; the higher the mark frequency, the smaller the spatial fluctuation. In underwater acoustic communication, the center frequency of the signal should be appropriately increased within the tolerance range of the high-frequency absorption and attenuation of the signal.

2. Simulation of signal interference at different horizontal distances

Simulation parameters: the seabed was absolutely hard and flat; water depth: 100 m; sound source deployment depth: 95 m (near the seabed); frequency: 5 kHz; mark bandwidth: 10 Hz; the horizontal distances of the transceiver end were 100 m, 300 m, and 1000 m.

**Figure 2:**
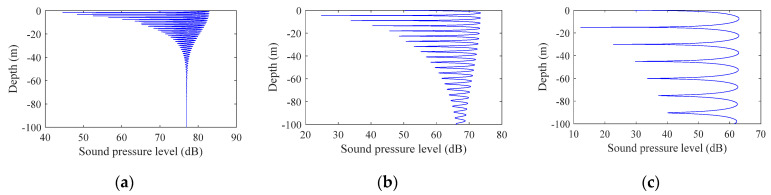
Signal interferograms at different horizontal distances: (**a**) 100 m, (**b**) 300 m, and (**c**) 1000 m.

Figure 2 shows the signal interference patterns at different horizontal distances. The distances from (a) to (c) were 100 m, 300 m, and 1000 m, where the abscissa is the mark sound pressure level and the ordinate is the depth. Comparing the figures, the range of vertical fluctuations in sound intensity increased exponentially as the horizontal distance increased, which was consistent with the inference obtained using Formula (11). In the case of the same bandwidth, as the signal frequency and the horizontal distance increased, the sound intensity fluctuation was inversely proportional to the signal frequency and proportional to the horizontal distance; as the depth increased, the sound intensity fluctuation decreased.

3. Interference simulation of different bandwidth signaling

Simulation parameters: the seabed was absolutely hard and flat; water depth: 100 m; sound source deployment depth: 95 m (near the seabed); frequency: 1 kHz; horizontal distance of the transmitting and receiving end 200 m; mark bandwidths were 1 Hz, 10 Hz, and 100 Hz.

**Figure 3:**
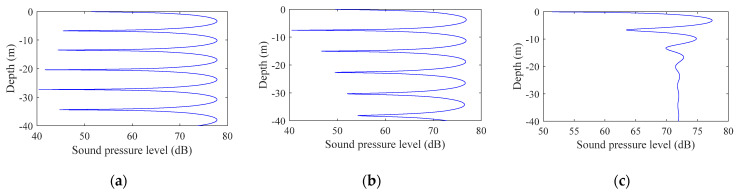
Signal interferograms at different bandwidths: (**a**) 1 Hz, (**b**) 10 Hz, and (**c**) 100 Hz.

Figure 3 shows the signal interference diagrams at different bandwidths. The bandwidths from (a) to (c) were 1 Hz, 10 Hz, and 100 Hz, where the abscissa is the signal sound pressure level and the ordinate is the depth. It can be seen from the comparison of the figures that as the bandwidth increased, the vertical fluctuation range of the sound intensity decreased exponentially until it tended to be stable in the end.

In summary, from the simulation results shown in Figure 1, Figure 2 and Figure 3, it can be seen that the signal fluctuation range was proportional to the distance between the sending and receiving ends and was inversely proportional to the signal center frequency, bandwidth, and modem lowering depth, which were related to Formulas (3)–(11) and were consistent with the inferences given.

#### 2.1.2. Analysis of Experimental Data

The bottom of the Yellow Sea is relatively flat and the sea conditions are relatively stable in the autumn, which is suitable for sound field analysis. With a view to verify the spatial distribution of the sound field obtained from the simulation experiments, a sound field analysis experiment ExQD_1701 was performed in the Yellow Sea in the autumn of 2017.

**Figure 4:**
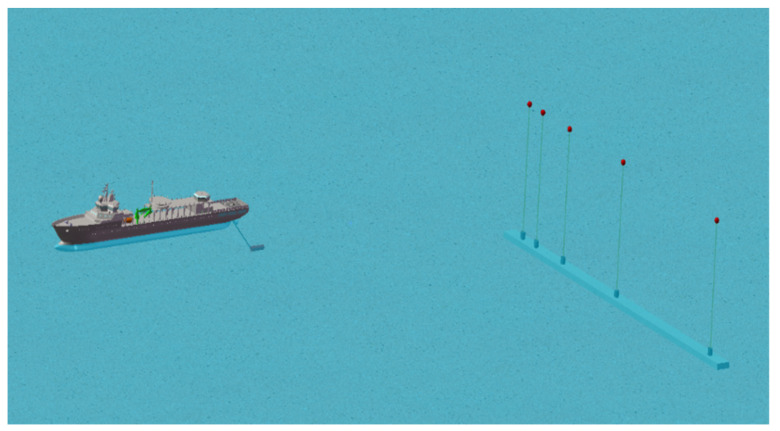
ExQD_1701 test.

The depth of this experimental sea area was precisely 40 m. A signal-launching ship, which used a UW350 type transmitting transducer with a working frequency range of 20 Hz–20 kHz, was used for the transmission. The transmitting transducer was cylindrical with a diameter of 0.2 m and a length of exactly 1 m. The net weight was exactly 100 kg with its own hoisting device. The schematic diagram of the experiment is shown in Figure 4. The Xiangyang Hong 81 experimental ship was utilized to be the signal-receiving ship with five sub-arrays of the same specification for signal reception with a pitch of 1 m. When the signal-transmitting ship reached the preset position, it could transmit signals with different frequencies. The real-time positions of the signal-transmitting ship and the signal-receiving ship were recorded using GPS, which was used to calculate the relative distance between the transmitting and receiving ends.

1. Spatial fluctuation of the low-frequency signal field

**Figure 5:**
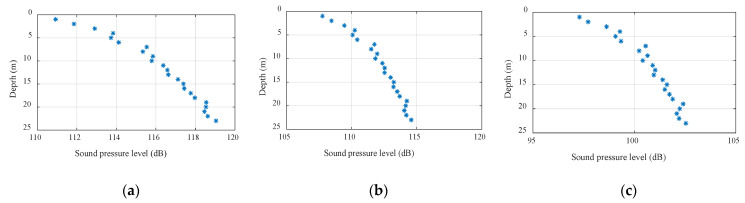
Spatial distribution at different distances (95 Hz): (**a**) vertical distribution at 1 km, (**b**) vertical distribution at 4 km, and (**c**) vertical distribution at 8 km.

**Figure 6:**
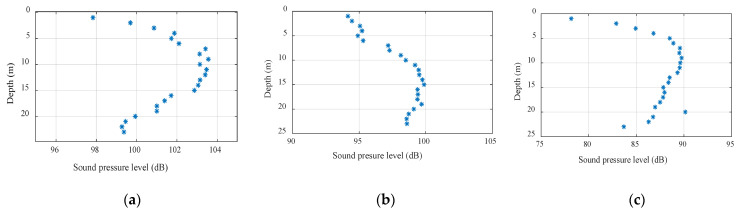
Spatial distribution at different distances (400 Hz): (**a**) vertical distribution at 1 km, (**b**) vertical distribution at 4 km, and (**c**) vertical distribution at 8 km.

The former simulation experiments demonstrated that when the signal frequency was low, the fluctuation was large. First, the experiment processed and analyzed the low-frequency signal data below 1 kHz. During the experiment, single-frequency signals of 95 Hz and 400 Hz were transmitted, and the sound source level of the transmitting transducer was stable. In order to summarize the spatial distribution of signals, Figure 5 and Figure 6 display the vertical distribution of sound pressure levels for single-frequency signals of 95 Hz and 400 Hz when the relative distances between the transmitting and receiving ends were different. The abscissa is the received sound pressure level and the ordinate is the water depth. By comparing different distances, frequencies, and depths, the spatial fluctuation characteristics of the signal could be summarized. When the signal frequency was low, the sea trial results fit well with the simulation results. As for the normal wave, the fluctuation law could be described as: the modes of the normal wave excited at different frequencies were different. The higher the frequency was, the greater the number of modes, and the more complicated the signal fluctuation law. This rule can be used to guide the equipment placement of low-frequency remote UAC systems.

2. Spatial fluctuation of the high-frequency signal field

**Figure 7:**
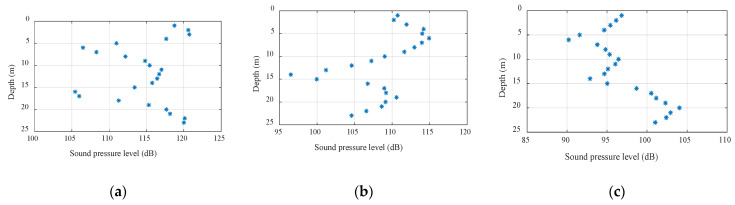
Spatial distribution at different distances (12 kHz): (**a**) vertical distribution at 1 km, (**b**) vertical distribution at 4 km, and (**c**) vertical distribution at 8 km.

**Figure 8:**
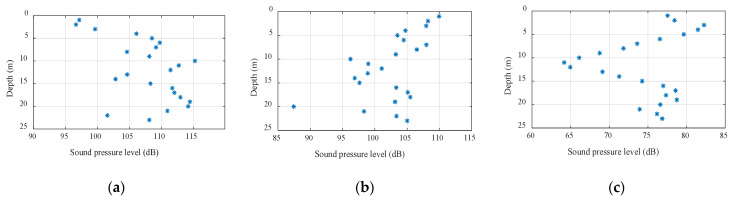
Spatial distribution at different distances (20 kHz): (**a**) vertical distribution at 1 km, (**b**) vertical distribution at 4 km, and (**c**) vertical distribution at 8 km.

The spatial fluctuations of high-frequency signals were also analyzed. The communication frequency band was selected from 5 kHz to 20 kHz with a steady energy level used in underwater acoustic communication experiments. The transmitting signals were single-frequency signals with frequencies of 12 kHz and 20 kHz. The sound source level of the transmitting transducer was stable. In order to discover the law of spatial fluctuations, Figure 7 and Figure 8 demonstrate the vertical distribution of the sound pressure level when the relative distances between the sending and receiving ends were different for single-frequency signals with frequencies of 12 kHz and 20 kHz, respectively. The abscissa in the figure is the obtained sound pressure level and the ordinate is the water depth. Through the comparison of different distances, frequencies, and depths, it was discovered that when the signal frequency was above 1 kHz, the signal wavelength was short; meanwhile, the environment was greatly affected during the propagation, which was difficult to study qualitatively. The farther the horizontal distance was, the larger the vertical fluctuation range, while the deeper the equipment deployment depth, the larger the vertical fluctuation range and the sound intensity of the near-sea surface signal was slightly lower than the signal strength in water. With the increase in frequency, the signal fluctuation is reduced; however, when the signal frequency was too high, i.e., the signal wavelength was short, which was greatly affected by the scattering and reflection of surface fluctuations, and the signal absorption loss was greater than when the frequency of the signal was low. Therefore, a single-frequency signal appeared to have a violent spatial distribution. When the frequency was as high as 20 kHz, there was a 25 dB intensity difference in the vertical distribution at a horizontal distance of 8 km.

### 2.2. Analysis of the Spatial Distribution of the Noise Field

**Figure 9:**
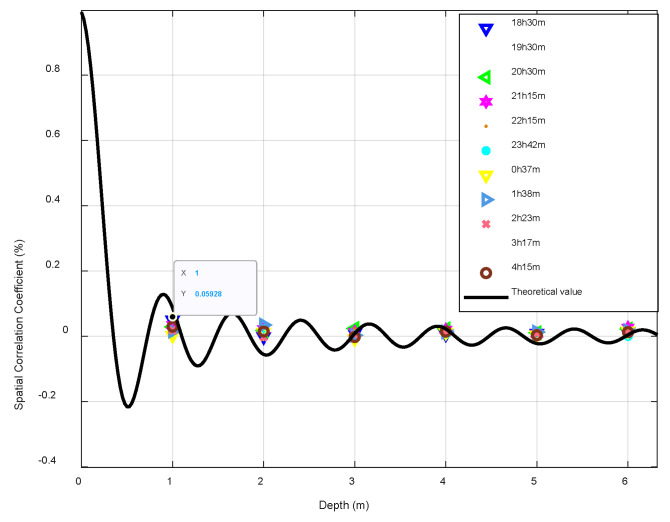
Correlation coefficient with depth (10 kHz, 10 h).

The time distribution characteristics of the noise field are mainly targeted at the commonly used high-speed underwater acoustic communication frequency bands of 5 kHz–20 kHz. According to the sound field analysis, in addition to the spatial distribution of the signal field due to the interference and the interface fluctuation, the influencing factors of the spatial variation range also have the spatial distribution of the system noise field. This study investigated the spatial distribution characteristics of the noise field in the 5 kHz–20 kHz frequency band with a general volume noise model [22]. It was assumed that all noise sources were uniformly distributed on an infinite plane; then, the spatial correlation coefficient of the marine environmental noise was simulated. In the ExQD_1701 experiment, the curve of the spatial correlation coefficient with respect to depth for a 10 kHz signal for 10 h is shown in Figure 9. The black dotted line is the theoretical value given by the general model of volume noise, and the spatial correlation of noise was small at high frequencies. Due to the correlation, calculations were performed between 30 array elements at different vertical depths and the no. 1 surface array element; consequently, the spatial correlation coefficient curve was clearly revealed. It was found that the curve matched the theoretical value given by the volume noise model. In subsequent high-frequency noise experimental data processing, the vertical correlation between array elements could be ignored.

**Figure 10:**
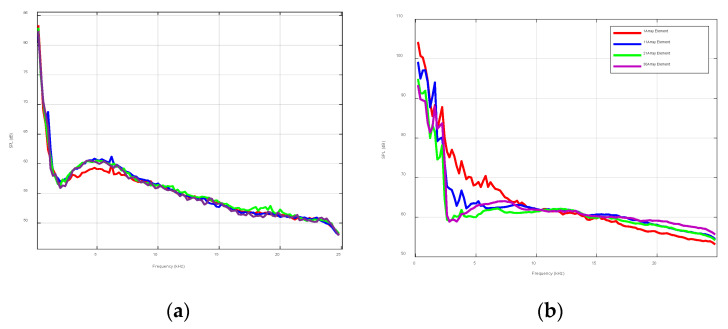
Environmental noise and vertical distribution of ship self-noise: (**a**) environmental noise and (**b**) ship noise.

In order to discuss the spatial distribution of wind and wave noise, the environmental noise was collected by using an array in ExQD_1701. The seabed in the experimental sea area was approximately the same level. The water depth was 40 m and the wind speed during the experiment was approximately 3 m/s. There were no other vessel activities within 5 km. Figure 10a indicates the noise field distribution of the marine environment at different depths, and Figure 10b shows the noise field distribution with the ship’s self-noise. The no. 1 array element was an offshore array element, and the no. 30 array element was a near-seabed array element. The ship’s self-noise had a greater impact on the frequency band below 5 kHz, which gradually decreased with the increase in depth and slightly fluctuated at high frequencies. Underwater acoustic communications often utilize high-frequency bands, with an associated 5 dB of noise fluctuations. The spatial distribution of environmental noise was not obvious and it was mainly because the surface noise source was a surface source composed of multiple noise sources. During the propagation process, the noise signals overlapped and neutralized each other with relatively small spatial fluctuation.

**Figure 11:**
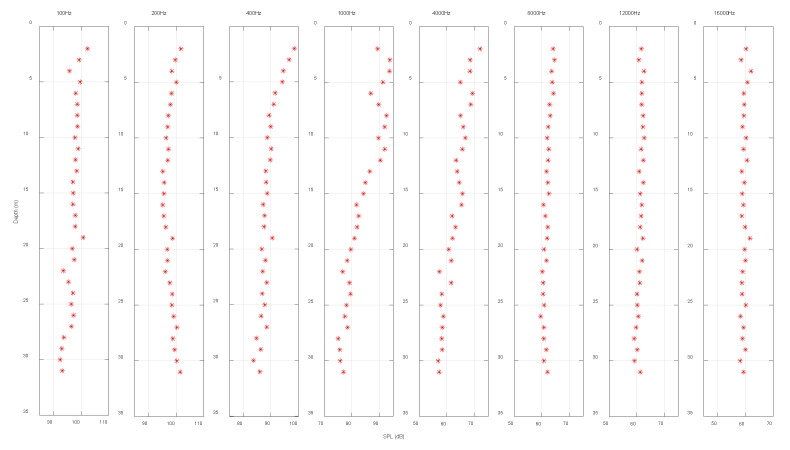
Different frequency noise field intensities with depth (40 m).

Figure 11 demonstrates the vertical distribution of noise at different frequencies measured using an array suspended from the side of the ship when the auxiliary ship was still working. Compared with the noise in Figure 10b, it can be considered that the noise was below 4 kHz. The noise in the frequency band was mainly the self-noise of the receiving ship. Due to this frequency band, the noise had a more obvious vertical distribution at each frequency point. As the depth increased, the noise power spectral density gradually increased, and the difference in noise spectral level could accumulate to approximately 20 dB. When the frequency was higher than 4 kHz, the noise mainly came from the surface waves, and the surface fluctuations contributed more significantly to the high-frequency noise field strength of 1 kHz–10 kHz. In the experiment, the array element closest to the surface was placed about 2 m underwater. When the water depth reached 5 m, the noise variation curve had no obvious fluctuations in the depth range covered by the hydrophone array. Thus, after the device was placed at a certain depth, the contribution of high-frequency noise to the fluctuation of the communication signal could be ignored.

According to the analysis of the experimental results, the spatial distribution of the signal field and the noise field was basically consistent with the simulation results, and its vertical distribution showed that the intensity of the near-sea surface signal was lower than that of other depth signals.

## 3. Analysis of the Time Fluctuation of Underwater Acoustic Communication Signals

The time window of a UAC system is smaller than other systems. Therefore, this study mainly analyzed the impact of small-scale spatial-temporal fluctuations caused by environmental parameters, such as wind and waves, on underwater acoustic communication. Moreover, environmental factors, such as ocean currents, tidal waves, and internal waves, with large spatial-temporal fluctuations were not considered.

### 3.1. Statistics of the Time Fluctuation of Low-Frequency Signal Fields

When an acoustic signal propagates in a shallow sea channel, it also has an undulating effect that changes over time, which corresponds to a time-varying channel in underwater acoustic communication. In this section, based on the spatial fluctuations of the signal field, the temporal fluctuations of the signal field were studied. The experimental ExQD_1701 data was statistically analyzed. In order to fully consider the selective frequency fading of the signal and ignore the effect of bandwidth on the time fluctuation of the signal field, the analysis used single-frequency signals. The time fluctuations in the low-frequency signal field and the high-frequency signal field were examined.

1. Spatial fluctuation of the high-frequency signal field

**Figure 12:**
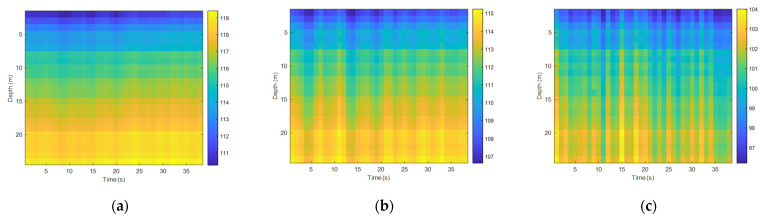
Temporal and spatial distribution at different horizontal distances (95 Hz): (**a**) spatiotemporal distribution map at 1 km, (**b**) spatiotemporal distribution map at 4 km, and (**c**) spatiotemporal distribution map at 8 km.

**Figure 13:**
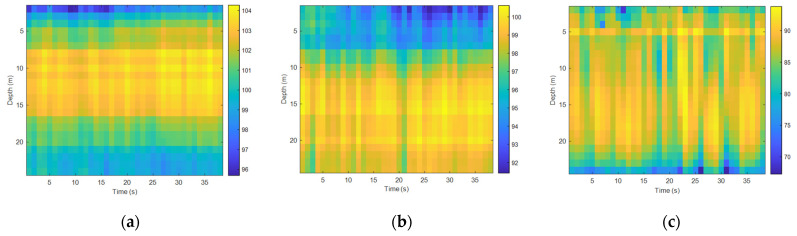
Temporal and spatial distribution at different horizontal distances (400 Hz): (**a**) spatiotemporal distribution map at 1 km, (**b**) spatiotemporal distribution map at 4 km, and (**c**) spatiotemporal distribution map at 8 km.

First, we analyzed the time fluctuation of the low-frequency signal. The frequencies of the transmitted signal were 95 Hz and 400 Hz, and the sound source level of the transmitted transducer was stable. Figure 12 and Figure 13 show the spurious color maps of the spatiotemporal distribution of the single-frequency signals (95 Hz and 400 Hz) at different distances. The vertical axis represents the water depth; the horizontal axis is the time when the signal was collected. With the increase in the horizontal distance between the transmitting and receiving ends, the signal strength increased significantly with time, which verified the conclusion of the theoretical calculations. The variation law of the signal intensity in the vertical direction was also consistent with the previous experimental results. When the frequency remained unchanged, the time fluctuation of the near-sea surface signal was larger than the sea floor fluctuation with the increase of the horizontal distance. With the increase in frequency, the number of normal wave modes of the signal field increased and the time fluctuation became stable.

2. Statistics of the time fluctuation of high-frequency signal fields

**Figure 14:**
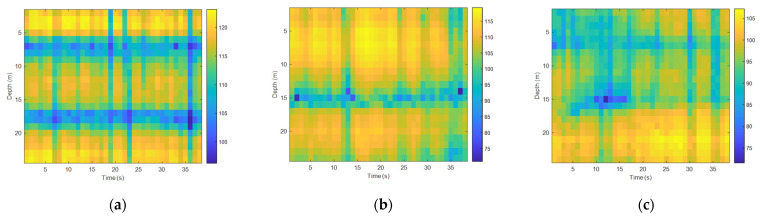
Temporal and spatial distribution at different horizontal distances (12 kHz): (**a**) spatiotemporal distribution map at 1 km, (**b**) spatiotemporal distribution map at 4 km, and (**c**) spatiotemporal distribution map at 8 km.

**Figure 15:**
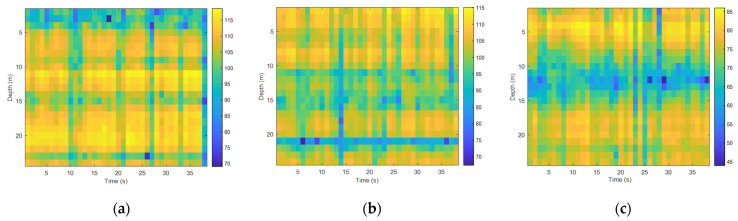
Temporal and spatial distribution at different horizontal distances (20 kHz): (**a**) spatiotemporal distribution map at 1 km, (**b**) spatiotemporal distribution map at 4 km, and (**c**) spatiotemporal distribution map at 8 km.

Analyzing the time fluctuations of high-frequency signals and selecting the 5 kHz–20 kHz communication frequency band were commonly used in underwater acoustic communication experiments, with the transmitted single frequency signals at 12 kHz and 20 kHz being used. The sound source level of the transmitting transducer was stable and it was the same as the processing flow of the time fluctuation of the low-frequency signal field. Figure 14 and Figure 15 show the spurious color maps of the spatiotemporal distribution of the single-frequency signals (12 kHz and 20 kHz) at different distances. The abscissa represents the time when the signal was collected, and the ordinate is the water depth, which indicates the distribution of the hydrophones from the surface to the sea floor. With the increase in distance, the time distribution of high-frequency signals became more pronounced, which showed that the channel structure stabilization time became shorter in underwater acoustic communication. As the frequency increased, the wavelength of the acoustic wave became shorter, the signal field became more complex under the influence of scattering and interference caused by interface fluctuations, and the fluctuation law of single-frequency signals appeared to be less significant. 

### 3.2. Analysis of the Time Distribution Characteristics of Noise Fields

**Figure 16:**
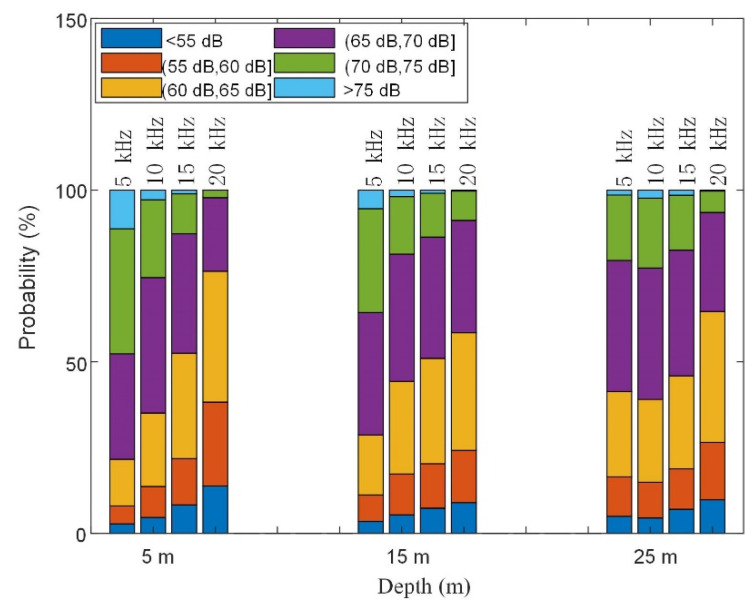
Noise distribution at different frequencies and different depths.

The time distribution characteristics of the noise fields were mainly targeted at the commonly used high-speed underwater acoustic communication frequency bands of 5 kHz–20 kHz. According to the foregoing, the vertical elements of the noise distribution were weakly correlated to each other. Therefore, the array elements at 5 m, 15 m, and 25 m were selected as research objects to study the noise time distribution in the surface, seabed, and water column situations. In the ExQD_1701 experiment, the environmental noise data in the experimental stage was randomly taken for 600 s, and a 1 s Hanning window was applied to intercept the data at an overlap rate of 0.66. Therefore, a total of 1762 sample points were obtained. Statistics of narrow-band noise distribution at different frequencies are shown in Figure 16. In the high-frequency range, the environmental high-frequency noise was mainly distributed in the 60 dB to 70 dB range. As the frequency increased, the noise intensity gradually decreased; the noise intensity also gradually decreased as the depth increased. This was found to be in accordance with the theoretical model.

In order to study the time distribution characteristics of noise further, the probability distribution of the single-frequency noise intensity at a depth of 15 m (Figure 16) was selected to explore the time distribution characteristics of noise.

**Figure 17:**
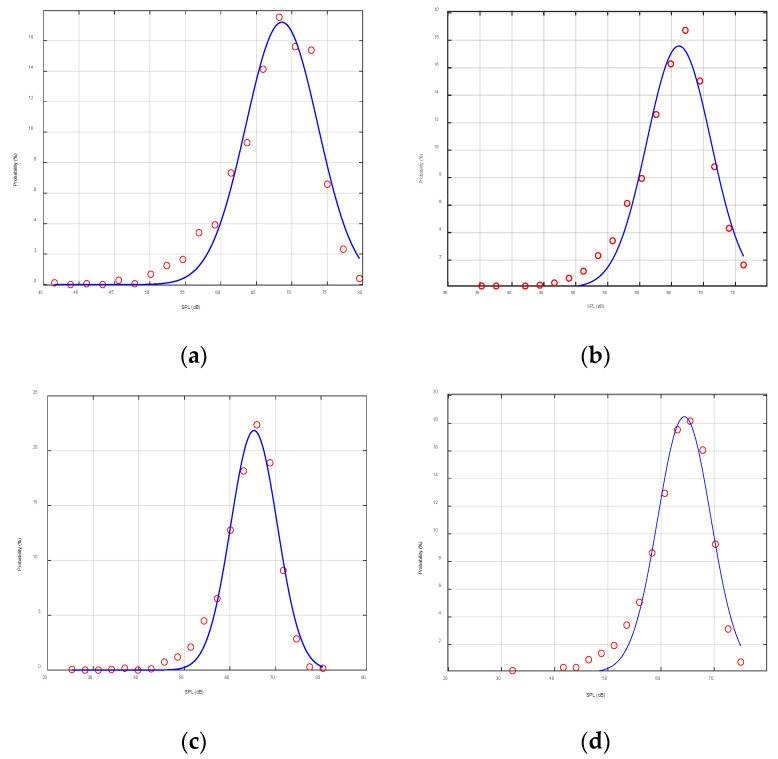
Statistical distribution of the noise over time: (**a**) 5 kHz, (**b**) 10 kHz, (**c**) 15 kHz, and (**d**) 20 kHz.

In Figure 17, subfigure (a) shows the time probability distribution of the 5 kHz narrowband noise with a mean of 68.59 dB and a standard deviation of 5.08 dB, subfigure (b) shows the time probability distribution of the 10 kHz narrowband noise with a mean of 66.16 dB and a standard deviation of 5.01 dB, subfigure (c) shows the time probability distribution of the 15 kHz narrowband noise with a mean of 65.30 dB and a standard deviation of 5.10 dB, and subfigure (d) shows the 20 kHz narrowband noise time probability distribution with a mean of 64.39 dB and a standard deviation of 4.97 dB. A comparison of these figures demonstrated that the probability of noise intensity basically followed the Gaussian distribution with a relativity small fluctuation on the time scale. As shown in Figure 17, the mean decreased with increasing frequency while the standard deviation remained stable.

According to the analyses of the above experimental results, it can be concluded that the time distribution of the high-frequency noise field was relatively stable. The analysis results can provide a theoretical guide and data support for the underwater acoustic communication quality evaluation model.

## 4. Analysis of the Spatiotemporal Variation Range of the Signal-to-Noise Ratio in Underwater Acoustic Communication

Based on the spatial-temporal distribution characteristics of the sound field obtained in the previous section, the spatial-temporal variation range of the SNR was analyzed from the experimental data. The data was selected from the Yellow Sea Acoustic Communication Experiment ExDQ_1702. The external field experimental parameters were: water with a depth of 15 m, communication signal frequency band of 8 kHz–16 kHz, transducer placement with a depth of 5 m, and five arrays for each receiving array. There were 32 hydrophones in each array and the distance between the hydrophones was exactly 0.5 m, the horizontal communication distance was 3.5 km, the experimental sea state was approximately two levels, and the vertical distribution of the entire bandwidth SNR was counted.

**Figure 18:**
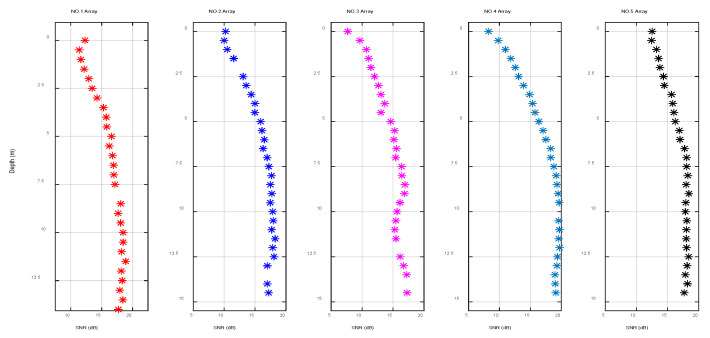
Signal-to-noise ratio of different arrays as a function of depth.

Figure 18 indicates the vertical fluctuation of the SNR of different arrays. Because the receiving array was limited by the size of the receiving ship, the relative horizontal distance between the arrays was not large and the gap between the SNR was small. During the underwater acoustic communication experiment, the auxiliary ship of the receiving ship was continuously working and displayed certain random fluctuations on the surface. As the receiving ship fluctuated up and down on the sea, the SNR of the surface array element was significantly lower than that of the underwater array element. With the increase in water depth, the variation range of the SNR gradually decreased and basically remained stable after 5 m underwater.

**Figure 19:**
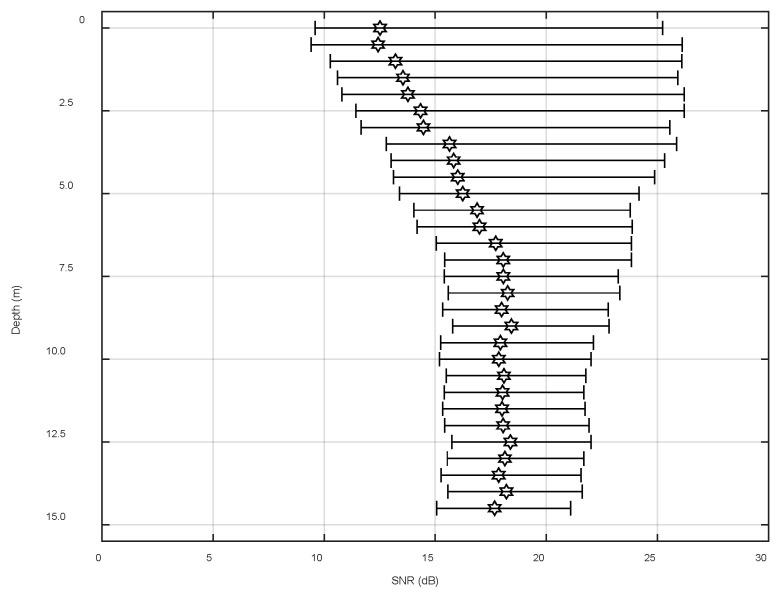
Signal-to-noise ratio fluctuation chart (fifth array).

**Figure 20:**
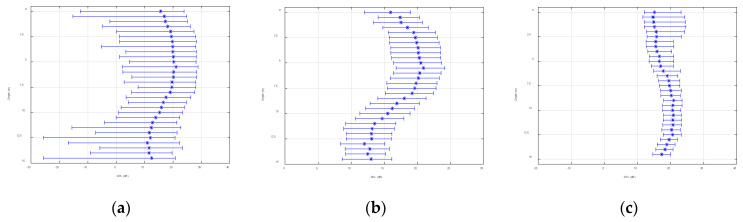
Signal-to-noise ratio vertical distribution (9 kHz): (**a**) bandwidth at 1 Hz, (**b**) bandwidth at 10 Hz, and (**c**) bandwidth at 500 Hz.

**Figure 21:**
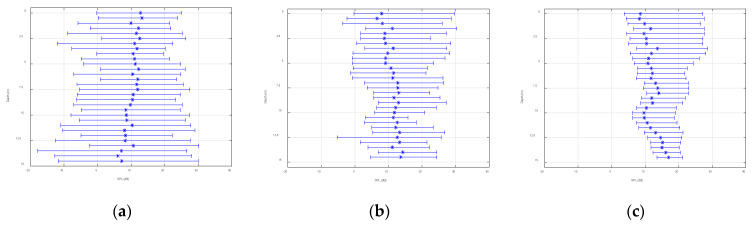
Signal-to-noise ratio vertical distribution (15 kHz): (**a**) bandwidth at 1 Hz, (**b**) bandwidth at 10 Hz, and (**c**) bandwidth at 500 Hz.

Figure 19 demonstrates the statistical range of the space-time variation of the SNR of array 5 over 31.5 s. The black hexagon indicates the mean of the SNR within the changing range, which is in line with Figure 18. From the figure, it was relatively small and fluctuated dramatically, which matched the trend of the spatial and temporal distributions of the signal field and noise field of the single-frequency signal. However, the experimental signal was further processed to cope with the slight inconsistency of the fluctuation range. Signals with center frequencies of 9 kHz and 15 kHz and a length of 31.5 s were selected. The sampling frequency of the system was 50 kHz and the number of sample points was about 1,574,520. The processing results are shown in Figure 20 and Figure 21. In these figures, the abscissa is the SNR and the ordinate is the depth, and the plots show the variation ranges of the SNR within the signal time of 31.5 s at different depths, with the blue point representing the mean value. With the increase in the processing bandwidth, the spatial-temporal variation range of the SNR was significantly reduced, and the SNR of the surface array elements in the vertical direction was significantly lower than that in the water array elements, which verified the laws obtained using the theory and simulation experiments.

**Figure 22:**
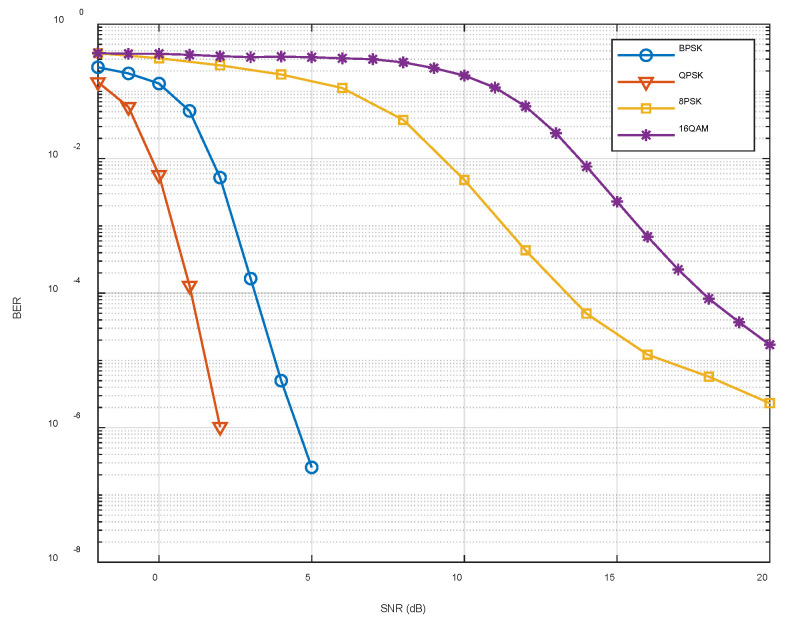
Bit error rate variation simulation results.

Figure 22 shows the simulation experiments performed under the fixed coding method using the channel parameters in the ExDQ_1702 experiment and the bit error rate corresponding to different signal-to-noise ratios (SNRs) and mapping methods. It can be seen that as the SNR increased, the bit error rate decreased. When the SNR in the experiment is less than 10 dB, the system should avoid using the 16QAM mapping method with a bit error rate higher than 0.01 and select other mapping methods according to the actual effective rate requirements.

## 5. Conclusions

Aiming at solving problems of the serious spatiotemporal fluctuations of the signal caused by the time-varying channel structure of the shallow sea, this study took identifying the spatiotemporal variation range of the SNR of underwater acoustic communication as the research goal, which was explored in the sound field from two aspects: the signal field and the noise field. The investigation of the temporal and spatial distribution of the signal field considered signal interference effects caused by surface reflection and scattering and theoretically deduced the variation of signal intensity fluctuations with horizontal distance, signal frequency, bandwidth, and deployment depth, which was further verified through both simulations and the Yellow Sea trial. The investigation of the noise field mainly considered the spatiotemporal distribution of high-frequency wind-induced noise and ship noise. Through processing and analysis of the experimental data, it was found that the time fluctuation of noise basically conformed to the Gaussian distribution, the spatial distribution consistency was high, and the near-sea surface noise was slightly higher than the bottom noise. Combining the analysis of the spatiotemporal distribution characteristics of the signal field and the noise field, it was found that when the signal frequency was high, the bandwidth was large; furthermore, when the horizontal distance between the transceivers was small and the depth of the receiver was deep, the spatial-temporal variation range of the SNR of the UAC system was relatively small. These characteristics were verified using sea trial data, and the derived law will be used to guide the parameter configuration and network protocol optimization of the UAC systems.

The correlation radius of the acoustic signal decreased with increasing frequency. The vertical correlation radius was smaller than the horizontal correlation radius. Therefore, in shallow sea acoustic communication, vertical arrays should be used for receiving to enhance the SNR and improve system reliability.

## Data Availability

Not applicable.
